# Kinetic control concept for the diffusion processes of paracetamol active molecules across affinity polymer membranes from acidic solutions

**DOI:** 10.1186/s13065-021-00794-7

**Published:** 2022-01-13

**Authors:** Sanae Tarhouchi, Rkia Louafy, El Houssine El Atmani, Miloudi Hlaïbi

**Affiliations:** Laboratoire Génie des Matériaux pour Environnement et Valorisation (GeMEV), Faculté des Sciences Ain Chock, Hasssan II University of Casablanca (UH2C), PB 5366, Maârif, Maroc

**Keywords:** Facilitated extraction, Affinity membranes, Permeability, Apparent diffusion coefficient, Association constant, Kinetic, And energetic controls

## Abstract

**Background:**

Paracetamol compound remains the most used pharmaceutical as an analgesic and antipyretic for pain and fever, often identified in aquatic environments. The elimination of this compound from wastewater is one of the critical operations carried out by advanced industries. Our work objective was to assess studies based on membrane processes by using two membranes, polymer inclusion membrane and grafted polymer membrane containing gluconic acid as an extractive agent for extracting and recovering paracetamol compound from aqueous solutions.

**Result:**

The elaborated membrane characterizations were assessed using Fourier-transform infrared spectroscopy (FTIR) and scanning electron microscopy (SEM). Kinetic and thermodynamic models have been applied to determine the values of macroscopic (***P*** and ***J***_**0**_), microscopic (D* and ***K***_***ass***_), activation and thermodynamic parameters (***E***_***a***_, ***ΔH***^***#***^, ***ΔS***^***#***^, ***ΔH***^***#***^_***diss***_, and ***ΔH***^***#***^_***th***_). All results showed that the PVA–GA was more performant than its counterpart GPM–GA, with apparent diffusion coefficient values (10^7^ D*) of 41.807 and 31.211 cm^2^ s^−1^ respectively, at T = 308 K. In addition, the extraction process for these membranes was more efficient at pH = 1. The relatively low values of activation energy (***Ea***), activation association enthalpy (***ΔH***^***≠***^_***ass***_), and activation dissociation enthalpy (***ΔH***^***≠***^_***di****ss*_) have indicated a kinetic control for the oriented processes studied across the adopted membranes much more than the energetic counterpart.

**Conclusion:**

The results presented for the quantification of oriented membrane process ensured clean, sustainable, and environmentally friendly methods for the extraction and recovery of paracetamol molecule as a high-value substance.

**Supplementary Information:**

The online version contains supplementary material available at 10.1186/s13065-021-00794-7.

## Introduction

In the last few decades, increasing attention has been paid to pharmaceutical industries that generate liquid wastes containing several pollutants and toxic substances [1–4]. These pollutants induce undesirable effects on the ecosystem and can potentially cause unexpected consequences and unintended effects on living organisms [5–8]. Consequently, treating these wastes becomes a major environmental issue for modern pharmaceutical industries and scientific research institutions. New technologies for the extraction, separation, and elimination of organic or inorganic substances and the recovery of several value-added molecules for evaluating these releases must be developed [9–12] to minimize and reduce the formation rate of toxic products [13].

Paracetamol is the raw material of many pharmaceutical products. Due to commercial and medical uses, modern industries use special methods to develop this active ingredient, which is not effectively removed by conventional methods during wastewater treatment. Thus, this pharmaceutical compound remains in municipal effluents, and different paracetamol concentrations have been detected in various parts of the world [14, 15]. Long-term exposure to drugs containing this active pharmaceutical ingredient can cause severe damage to humans and other animals [16–19]. Therefore, its recovery and extraction from industrial liquid waste is the need of the hour.

In recent years, various membrane processes have been reported for various applications [20–22] (such as removal, purification, recovery, and extraction of organic compounds present in liquid wastewater). Membrane-based technology has become critical and has attracted much attention as a valuable technology for many industries due to the distinctive capability of selective and efficient extraction of target species (e.g., ions/small molecules). It is an environmentally friendly alternative that considerably reduces the volume of used chemical products, and minimal energy is consumed during the process. These methods are successfully applied in several fields, such as environment, energy, health, water treatment, cosmetic, food, chemical, and pharmaceutical industries. Depending on their structure, composition, and morphology, a wide range of membranes (including organic polymer membranes) can be developed for use in different fields. These favorable properties and functionalities exhibit clear and important advantages compared to other separation and extraction techniques such as resin separation, liquid–liquid extraction (ELL), solid-phase extraction (EPS), and chromatography [23–27]. These properties help in determining the selectivity parameters in particular.

In general, the extraction mechanism through a membrane is based on facilitated diffusion. These oriented membranes that promote facilitated extraction are now the subject of several studies. Facilitated extraction membranes employ chemicals (hereafter denoted extractive agent) to specifically and reversibly react with the target spices to form (Substrate-Extractive agent) complexes, then transport the complexes from the feed phase to the receiving phase allowing the regeneration of the substrate via reverse reactions. The separation efficiency of the facilitated extraction through the polymer membranes is principally governed by reaction kinetics at membrane/aqueous solution interfaces, together with the extraction rate of the complexes (Substrate-Extractive agent) through the membrane matrix. According to the extraction agent mobility and physicochemical properties of the facilitated extraction membrane, an extraction mechanism based on the successive jump of the substrate via a semi-mobile and fixed extractive agent has been proposed [28]. The studied membranes polymer inclusion membranes (PIMs), and grafted polymer membranes (GPMs), as two major types of facilitated extraction membranes, have attracted much attention in fundamental studies and practical applications [29–32]. Due to the simple preparation steps, stability, better mechanical properties, good chemical resistance, better mechanical properties, and particularly stable integration of the extractive agent into the polymer support, special attention is paid to PIMs and GPMs [33–36].

This study highlights the development of a clean and sustainable treatment process in the pharmaceutical industry. Accordingly, in our laboratory, experiments related to the facilitated extraction process of paracetamol, which is used here as a model drug to evaluate the extraction capabilities of the membrane process, were carried out to extract the active substances from the liquid solution. Our challenge was to determine the proper and selective extractive agent and examine its effectiveness in developing a stable and efficient membrane for extracting paracetamol. We also aimed to evaluate the parameters to achieve high recovery, high throughput, and low consumption time. This extraction process was performed using **PIM** and **GPM** containing gluconic acid (**GA**) as the extractive agent. The prepared membranes were characterized by two techniques: (i) Fourier-transform infrared spectroscopy and (ii) scanning electron microscopy (SEM) techniques to confirm the presence of the extractive agent in the polymeric support. The developed membranes were used to perform oriented processes for facilitated extraction and to recover paracetamol substrates under the influence of the initial substrate concentration, acidity, and temperature of the medium. The dynamics of mass transfer and the effect of the different factors on the extracting the paracetamol substrate were discussed. The kinetically determining step, which controls the rate of paracetamol extraction when PIMs and GPMs are used, has been elucidated by analyzing the kinetic data.

## Methods/experimental

### Chemicals and reagents

Paracetamol was purchased from ICN Biomedicals. All polymers, polyvinyl alcohol (PVA) (Mw = 72,000 g mol^−1^), polysulfone (PSU) (Mw = 35,000 g mol^−1^), polyvinyl-pyrrolidone (PVP) (Mw = 45,000 g mol^−1^), GA (Mw = 218, 2 g mol^−1^), and the solvent *N*,*N*-dimethylformamide (DMF, 99.8%) and dimethylsulfoxide (DMSO ˃ 99.8%), are commercial products (Aldrich, Fluka). Double distilled water was used in all experiments. The pH of aqueous solutions was adjusted with an analytical grade solution of hydrochloric acid (HCl) from Sigma.

### Instruments and apparatus

The acidities of the aqueous solutions (feed phase and receiving phase) were measured using a pH meter (HANNA Instruments HI 8519N). A UV–visible spectrophotometer (Rayleigh. U.V.—2601) was used, to determine the paracetamol concentration (C_R_) in the receiving phase. Two infrared spectrophotometers, AVATAR 360 FTIR ESP and JASCO model 4600 were used to plot the FTIR spectra to identify the presence of extractive agents in the polymer matrix. Similarly, the scanning electron microscopy (SEM) technique was used to produce different micrographs and study the morphology and porosity of the developed membranes by (ZEISS EVO40 EP) and (JEOL NeoScope JCM-500). Their thicknesses were measured using an electronic micrometer (Mitutoyo).

### Membrane preparation

To conduct the oriented processes of the facilitated extraction of paracetamol, we have prepared two types of polymer membranes **PIM** and **GPM**, based on polyvinyl alcohol and polysulfone as polymer support with the same extractive agent (**GA**).

The adopted **GPM** was developed according to the following experimental protocol [37]: a 3 g of polysulfone polymer dissolved in 13 cm^3^ of dimethylformamide (DMF) was introduced into a closed bottle to isolate the mixture from the air. The system was stirred for 12 h until polysulfone was completely solubilized. Next, a 0.625 g of polyvinylpyrrolidone (PVP) was added to this homogeneous solution, followed by the slow addition of an equivalent mass of 3 × 10^−3^ mol of GA. The mixture was stirred for 3 to 4 days to solubilize the extractive agent to produce a homogeneous phase. The resulting phase was cast on a glass plate and then spread with a ruler. This glass plate was rapidly immersed in a bath containing distilled water. The solvent DMF leaves the membrane matrix and a rigid membrane in the form of a paper (phase inversion method) was obtained [38, 39]. After this operation, the GPM membrane was dried, its mass (0.030 g) and thickness (***l*** = 162 µm) were determined. Its total surface area (10 cm^2^) was measured and the concentration of the extractive agent [**T**]_**0**_ = 0.20 mol L^−1^ was calculated.

**PIM** [40] was prepared by dissolving 10 g of polyvinyl alcohol (PVA) in a mixture of 20 cm^3^ of DMSO and 80 cm^3^ of distilled water. The mixture was stirred for 24 h at a temperature of 120 °C to dissolve the PVA in the solution. In this homogeneous solution, an equivalent mass of 3 × 10^−3^ mol of GA was added slowly under a condition of constant stirring to avoid polymer aggregation. The resulting solution was poured carefully into a Petri dish and placed on a stove at a temperature range of 70 to 80 °C to evaporate the solvent completely. The heating temperature promotes solvent evaporation, allowing the polymer and extractive agent chains to come together. This step is important in the PIM membrane development process. The approach facilitates the cross-linking between the extractive agent and the polymer, inducing faster cross-linking kinetics [41]. The PIM obtained by this experimental protocol (heat vulcanization method) was homogenous, transparent, flexible, and mechanically strong [42, 43]. Its thickness was measured (***l*** = 228 µm) and the extractive agent concentration was calculated ([**T**]_**0**_ = 0.30 mol L^−1^).

### Experimental protocol for the facilitated extraction of paracetamol

The experimental cell (Additional file 1: Fig. S1) was used to carry out the facilitated extraction processes of the paracetamol compound. It consists of two compartments of identical volume separated by the produced membrane. The feed phase (**F**) contained the paracetamol solution in the concentration range of 0.01 to 0.08 (mol L^−1^), and the receiving phase (**R**) contained distilled water [40, 44]. The aqueous phase volume was 70 cm^3^ in each compartment. The system was immersed in a thermostatic bath (**TB**) containing water to keep the temperature constant throughout the experimental procedure. Homogeneity was ensured by using a multi-station magnetic stirrer.

Samples were collected from the receiving phase every 30 min and were measured at absorption maximum wavelength (ƛmax = 244 nm). Knowing these values is necessary to calculate the membrane volume to determine the fixed concentration [**T**]_0_ of GA in the membrane phase.

## Results and discussion

Before adopting these membranes for the facilitated extraction process of paracetamol substrate under different experimental conditions, various studies on their compositions and their morphologies were performed.

### Fourier transform-infrared (FTIR) analysis

**Fig. 1:**
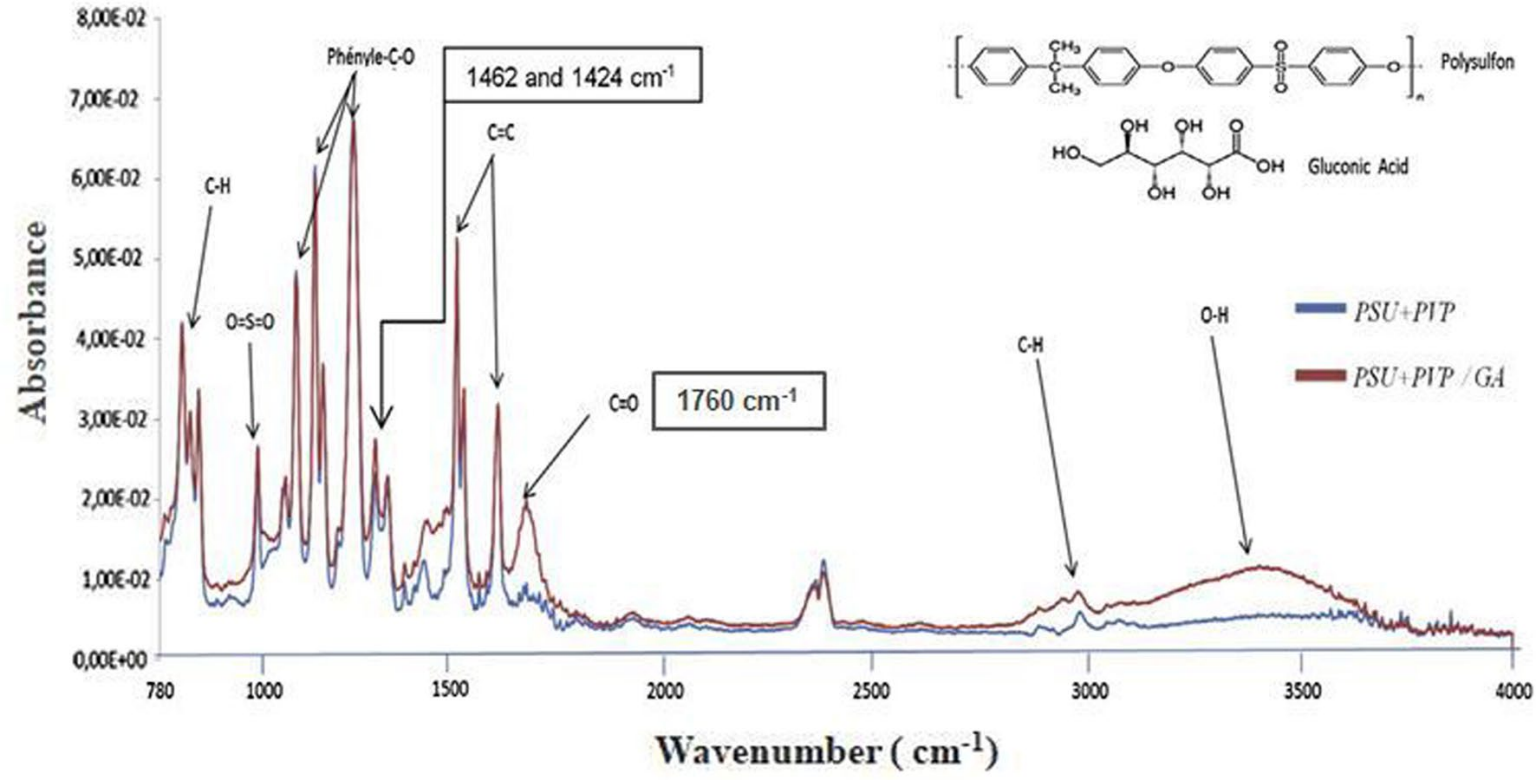
The FT-IR spectrum for the PSU + PVP support and the PSU/PVP–GA membrane

After drying the sample for 48 h to remove traces of residual water and solvent, the obtained membranes (PSU–PVP) and (PSU–PVP–AG) were characterized by the FTIR-spectroscopy technique (Fig. 1) to record the vibration bands corresponding to the membrane components. The PSU–PVP/GA membrane spectrum shows that all the characteristic absorption bands of the PSU + PVP support are present. These FT-IR spectra related to the PSU + PVP support membrane show the peaks existing in the range of 700–1400 cm^−1^ correspond to PSU fingerprints, and two vibration peaks (1462 and 1424 cm^−1^), corresponding to the tertiary amine group of PVP copolymer. The spectrum also indicates the presence at around 3200–3600 cm^−1^ of a characteristic broad absorption band corresponding to the alcohol group (OH). A peak at 1720 cm^−1^ was also observed, which was attributed to the vibration of the C=O group of GA. These spectral evolutions proved that the extractive agent GA has been successfully integrated into the polymer matrix of the membrane.

The PIM was analyzed and characterized using the FT-IR and SEM in the same manner as the membrane described in the previous section. The results indicated and proved that the extractive agent; was trapped in the polymer matrix of the membrane, whose porosity increased with the concentration of the extractive agent.

**Fig. 2:**
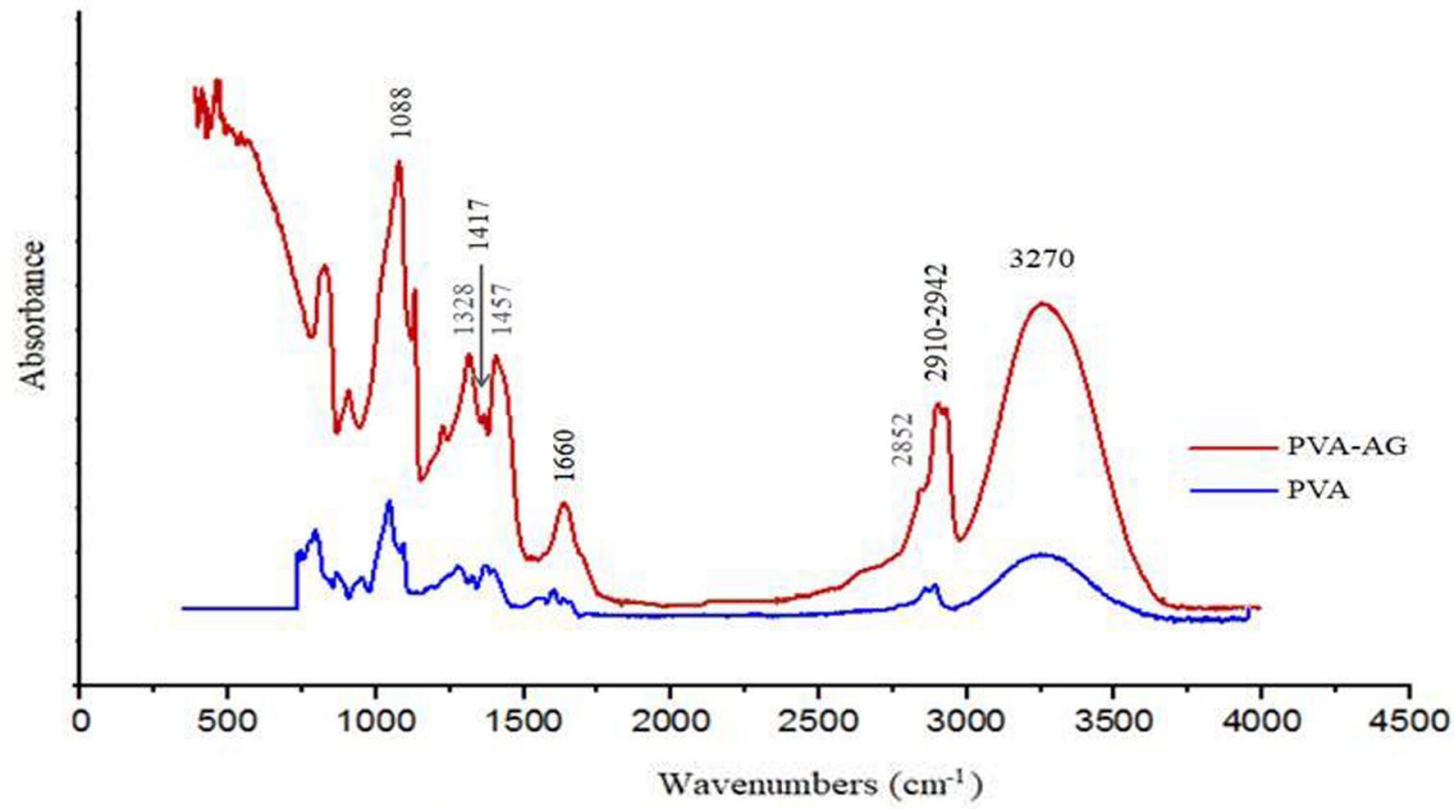
The FT-IR spectrum for the PVA support and the PVA–GA membrane

Figure 2 shows the FTIR spectra of the PVA support and PVA–AG membranes. The common stretching vibration bands for some relative wavenumbers of the PVA polymer are: from 3283 to 3400 cm^−1^ attributed to the OH stretching vibration; from 2850 to 3000 cm^−1^ associated with the asymmetric stretching vibration of CH_2_ or CH; the bands 1327 and 1424 cm^−1^ are due to the bending vibrations of CH_2_ and CH_3_. It is expected that the inclusion of AG agent in the PVA support increases the number of hydroxyl groups. As a result, the absorbance intensity band for –OH increases, and a new slightly intense peak for the vibration of the C=O (carboxylic) bond appears at 1660 cm^−1^. A homogeneous dispersion of the extractive agent in the polymer matrix has a crosslinking effect due to covalent bonds’ formation involving chemical interactions between polymer functional groups and organic acids at a high temperature [45, 46]. On the other hand, several crosslinking methods have been published for different uses, since as a rule, all multifunctional compounds capable of reacting with hydroxyl groups can be used to obtain tridimensional networks in PVA [47, 48]. In addition, Heat-treatment above the glass transition temperature is also used as means of achieving the same results [49, 50].

### Scanning electron microscopy (SEM) analysis

**Fig. 3:**
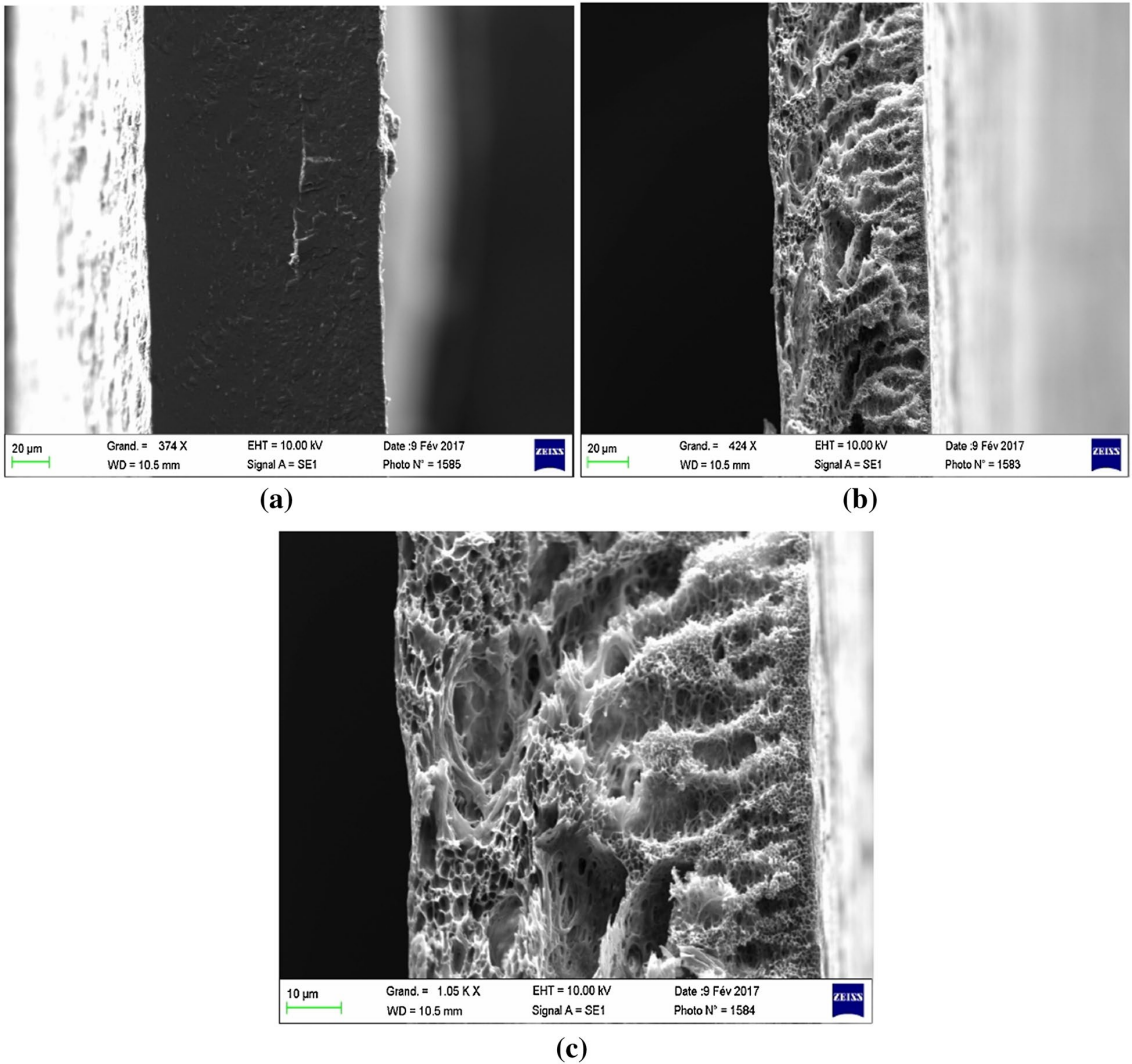
SEM micrographs: **a** support polymer cross-section (PSU/PVP), **b**, **c** membrane cross-section (PSU/PVP–GA)

Various samples of the elaborated membranes were visualized using the SEM technique. The samples were irradiated with an electron beam (15 kV). This study was carried out under suitable magnification. Electrons were precisely focused for better visualization of the membrane surface and to properly record SEM micrographs of the upper surface of the polymer support (PSU + PVP) and the GPM membrane (PSU + PVP + GA). SEM images of the membranes with different compositions are grouped in the scheme of Fig. 3.

The SEM micrograph, presented in Fig. 3a, represents the morphology of the polymer support (PSU-PVP). A considerably smooth and dense surface without apparent porosity was observed. Figure 3b, c reveal that the extractive agent was efficiently grafted onto the membrane phase. It also influenced the structure, morphology, and porosity of the polymeric support. The synthesized membrane contained pores along the membrane width (surface layers; Fig. 3b, c.

**Fig. 4:**
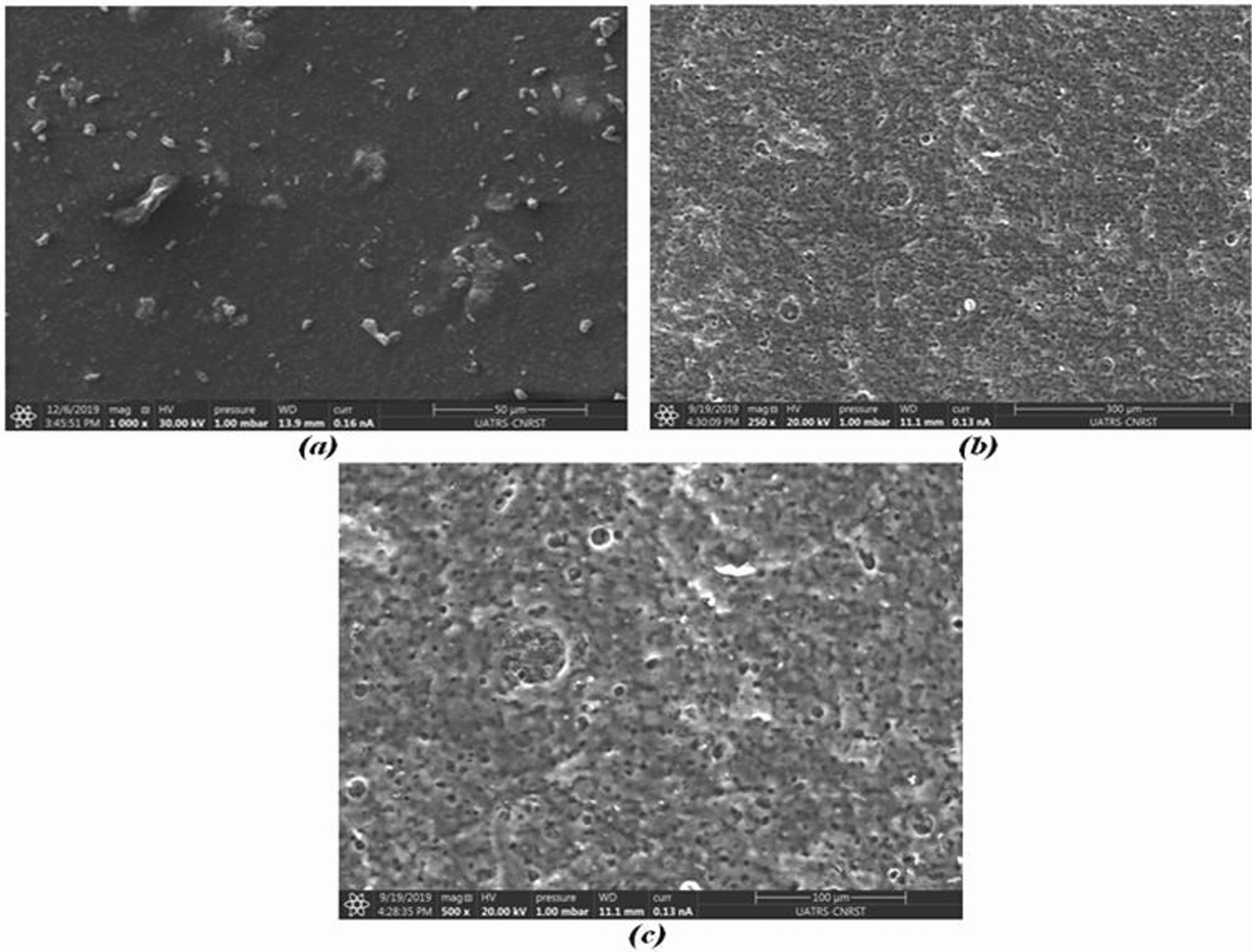
SEM micrographs of support polymer surface (PVA) (**a**) and membrane surface (PVA–GA) (**b**)

Figure 4 represents the SEM images relative to the two prepared PVA and PVA–AG. These SEM micrographs generally showed a remarkable change in morphology and porosity with the inclusion of the extractive agent in the polymeric support. Image (a) corresponds to the surface of the PVA support and clearly shows that the surface is homogeneous and smooth without apparent porosity. In contrast, the membrane modified by the inclusion of gluconic acid exhibits a clear porous membrane structure with a largely homogeneous porosity (b, c) which are included in the polymer matrix.

### Degree of swelling

The degree of swelling versus time was investigated by measuring the change in weight of the membrane before and after the swelling. The different sample membranes of 3 × 3 cm were immersed into distilled water at pH = 1, 2 and 3 for 48 h. The membranes were taken out from the water every time tx, and carefully wiped with an absorbent paper, and quickly weighed. Increase in weight of the film was determined at preset time intervals until a constant weight was observed. The experiments were performed in triplicate, and average values were reported. The degree of swelling was calculated using the following equation [51, 52]:

$${\text{DS}}\left( \% \right) \, = \frac{{{\text{W}}_{{\text{t}}} - {\text{W}}_{0} }}{{{\text{W}}_{0} }} \times 100,$$

where W_t_ is the weight of film at time t, and W_0_ is the weight of film at time zero, and the value of W_t_ is the result of the average of three weighings for each membrane.

Additional file 1: Figure S2 depicts the degree of swelling of the membranes PIM-based PVA and PIM–GA at pH = 1, 2 and 3. The results reveal that the pH of the medium doesn't play a role in affecting the swelling of membranes PIM–GA and improves mechanical properties [53, 54]. In addition, PIMs cross-linked by the GA have DS ≤ 23.5% compared to the membrane-based PVA only DS < 52%. These confirm that the cross-linking effect with GA reduces the swelling degree. The efficiency of cross-linking and swelling ratio of the membranes are the main parameters to define its physicochemical properties.

The same experiment was conducted for GPM, and the membrane maintains practically the same weight after immersing in distilled water. The membrane has a well-aligned layer structure that does not swell. This result is probably due to the reason the polymer properties (hydrophobic), and the crosslinking type play an important role in effectively stabilizing the membrane and preventing them from swelling. Furthermore, this result was explained by Gupta et al. [55] that considered that the crosslinking factor influences the swelling behavior and hence the resistivity of the membranes. The higher resistivity in both the 2 and 4% cross-linked membranes for higher graft levels is therefore due to the lower water content as observed in the swelling behavior.

### Theoretical models for quantification of processes

The facilitated extraction processes for substrate **S** were conducted using an affinity polymer membrane. The process depends on the association and the dissociation of the substrate-extractive agent entity (**ST**) at the membrane-solution interfaces and in the membrane phase during the substrate diffusion. To quantify the processes carried out and to study the performances of the adopted membranes, kinetic and thermodynamic models based on the first and second Fick’s laws and a saturation law of the extractive agent (**T**) by the substrate (**S**) have been developed in the laboratory [37, 40, 56–58]. The equilibrium “association/dissociation” is presented according to the following relationships.

1 $${\varvec{P}} \times \left( {{\mathbf{t}} - {\mathbf{t}}_{{\mathbf{I}}} } \right) = \left( {{\varvec{l}} \times {{\text{V}} \mathord{\left/ {\vphantom {{\text{V}} {\text{S}}}} \right. \kern-\nulldelimiterspace} {\text{S}}}} \right)\left[ {1/2 \times Ln\left( {{\mathbf{C}}_{{\mathbf{0}}} /{\mathbf{C}}_{{\mathbf{0}}} - 2{\mathbf{C}}_{{\mathbf{R}}} } \right)} \right],$$

2 $${\varvec{J}}_{{\mathbf{0}}} = \left( {{\varvec{D}}^{\user2{*}} /{\varvec{l}}} \right) \times \left[ {\left[ {\varvec{T}} \right]_{{\mathbf{0}}} \times {\varvec{K}}_{{{\varvec{ass}}}} \times {\mathbf{C}}_{{\mathbf{0}}} /\left( {{\mathbf{1}} + {\varvec{K}}_{{{\varvec{ass}}}} \times {\mathbf{C}}_{{\mathbf{0}}} } \right)} \right].$$

***l***: membrane thickness (cm), **S**: membrane active area (cm^2^) and **V**: receiving phase volume (cm^3^).

**C**_**0**_, **C**_**R**_, and [**T**]_**0**_: initial substrate concentration in the feed phase (mol L^−1^), substrate concentration in the receiving phase at time ***t*** (mol L^−1^) and extractive agent concentration in the organic phase (mol L^−1^), respectively.

***P***: membrane permeability (cm^2^ s^−1^), ***J***_**0**_: substance initial flux across the membrane (mmol s^−1^ cm^−2^), ***K***_***ass***_: association constant of entity **ST** (L mol^−1^), and D*: apparent diffusion coefficient of the substrate S through the membrane phase (cm^2^ s^−1^).

If the kinetic model is verified, after an induction time (**t**_**I**_), the function (− Ln (**C**_**0**_ − 2**C**_**R**_) versus time) evolves linearly. The slope (***a***) of the obtained straight line allows the determination of the permeability parameter ***P*** according to the following equation [59, 60].

3 $${\varvec{P}} = \left( {{\varvec{a}}*{\varvec{V}}*{\varvec{l}}} \right)/{\mathbf{2}}{\varvec{S}},$$

The initial flux ***J***_**0**_ can be calculated from the permeability coefficient ***P*** by the following equation:

4 $${\varvec{J}}_{{\mathbf{0}}} = \left( {{\varvec{P}} \times {\varvec{C}}_{{\mathbf{0}}} } \right)/{\varvec{l}}.$$

To determine the nature of the movement of the substrate S during its diffusion through the membrane phase and to elucidate the mechanism that governs the studied processes, it is necessary to determine the values of the microscopic parameters D* and ***K***_***ass***_. We used the Lineweaver–Burk method (L–B) to linearize the expression in Eq. 2, according to the following equation [44, 61]:

5 $${\mathbf{1}}/{\varvec{J}}_{{\mathbf{0}}} = \left( {{\varvec{l}}/{\varvec{D}}^{\user2{*}} } \right) \times \left[ {\left( {{\mathbf{1}}/\left( {\left[ {\mathbf{T}} \right]_{{\mathbf{0}}} \times {\varvec{K}}_{{{\varvec{ass}}}} } \right)} \right) \times \left( {{\mathbf{1}}/{\mathbf{C}}_{{\mathbf{0}}} } \right) + \left( {{\mathbf{1}}/\left[ {\mathbf{T}} \right]_{{\mathbf{0}}} } \right)} \right].$$

The linear evolution of the term 1/***J***_**0**_ = f (1/**C**_**0**_) (from Eq. 5) allows us to confirm that the thermodynamic model is based on the interaction of the substrate (**S**) with the extractive agent (**T**). The interaction in the membrane phase was checked. Similarly, the values of slopes (**p**) and intercepts (**OO**) of the obtained straight line segments are used to calculate the values of D* and ***K***_***ass***_ according to the following equation:

6 $${\varvec{K}}_{{{\varvec{ass}}}} = {{{\varvec{intercept}}\left( {{\varvec{OO}}} \right)} \mathord{\left/ {\vphantom {{{\varvec{intercept}}\left( {{\varvec{OO}}} \right)} {{\varvec{slope}}}}} \right. \kern-\nulldelimiterspace} {{\varvec{slope}}}}\;{\text{and}}\;{\varvec{D}}^{\user2{*}} = \left( {{\varvec{l}}/{\varvec{OO}}} \right) \times \left( {{{\mathbf{1}} \mathord{\left/ {\vphantom {{\mathbf{1}} {\left[ {\mathbf{T}} \right]_{{\mathbf{0}}} }}} \right. \kern-\nulldelimiterspace} {\left[ {\mathbf{T}} \right]_{{\mathbf{0}}} }}} \right).$$

The initial flux is related to the temperature factor by the Arrhenius law [62, 63], according to the following equation:

7 $${\varvec{J}}_{{\mathbf{0}}} \left( {\varvec{T}} \right) = {\varvec{A}}_{{{\varvec{j}} }} {\varvec{exp}}\left( { - {\varvec{E}}_{{\varvec{a}}} /{\varvec{RT}}} \right),$$

***R***: gas constant (8.314 J mol^−1^ K^−1^). ***A***_***j***_: proportional term to the favorable interactions (mol^−1^ s^−1^ m^2^), ***E***_***a***_: transition state activation energy of the formation-dissociation reaction of the entity (***TS***) (J mol^−1^).

The expression was linearized according to the following equation:

8 $${\varvec{lnJ}}_{{\mathbf{0}}} = \left( {\left( {\left( { - {\varvec{E}}_{{\varvec{a}}} } \right)/{\varvec{R}}} \right) \times \left( {{\mathbf{1}}/{\varvec{T}}} \right) + {\varvec{lnA}}_{{\varvec{j}}} } \right).$$

The values of activation parameters ***E***_***a***_ and ***Aj*** were determined from the slope and the intercept of the linear function Ln (***J***_**0**_) = f (1/**T**). According to the transition state theory (Eyring theory), these values allow the calculation of the activation enthalpy ***ΔH***^***#***^ (J mol^−1^) and entropy ***ΔS***^***#***^ (J K^−1^ mol^−1^) parameters from the following equation:

9 $$\user2{\Delta H}^{\user2{ \ne }} = {\varvec{E}}_{{\varvec{a}}} - 2500 \left( {{\text{J}}\;{\text{mol}}^{ - 1} } \right)\;{\text{and}} \;\user2{\Delta S}^{\user2{ \ne }} = {\varvec{R}}\left( {{\varvec{lnA}}_{{\varvec{j}}} - 30.46} \right) \left( {{\text{J}}\;{\text{K}}^{ - 1} \;{\text{mol}}^{ - 1} } \right)\;{\text{at}}\;298\;^\circ {\text{K}}{.}$$

The thermodynamic enthalpy parameter ***ΔH***^***≠***^_***th***_ (Kj mol^−1^) represents the amount of energy exchanged during the equilibrium reaction related to the formation of the **ST** entity. The value of this parameter is determined directly from the slope of the linear representation of Van’t Hoff’s law (Eq. 10).

10 $${\mathbf{ln}}\left( {{\varvec{K}}_{{{\varvec{ass}}}} } \right) = ( - (\user2{\Delta H}_{{t{\varvec{h}}}}^{\user2{ \ne }} )/{\mathbf{RT}}) + {\varvec{cste}}.$$

On the other hand, according to the transition state theory, for an elementary reaction, this important thermodynamic parameter is related to the activation enthalpies, association ***ΔH***^***≠***^_***ass***_, and dissociation ***ΔH***^***≠***^_***diss***_ (Kj mol^−1^) by the following relation:

11 $$\Delta {\varvec{H}}_{{{\varvec{th}}}}^{ \ne } = \Delta {\varvec{H}}_{{{\varvec{ass}}}}^{ \ne } - \Delta {\varvec{H}}_{{{\varvec{diss}}}}^{ \ne } .$$

### Influence of the initial substrate concentration (C_0_) on the performance of the developed membranes

Before adopting PIM–GA and GPM–AG, we have carried out experiments related to the extraction of paracetamol through PVA and PSU–PVP membranes without the extractive agent. We have noticed that these membranes are impermeable and confirm that the extractive agent is essential, which is responsible for interactions with the target species and their diffusion through the membrane phase.

**Fig. 5:**
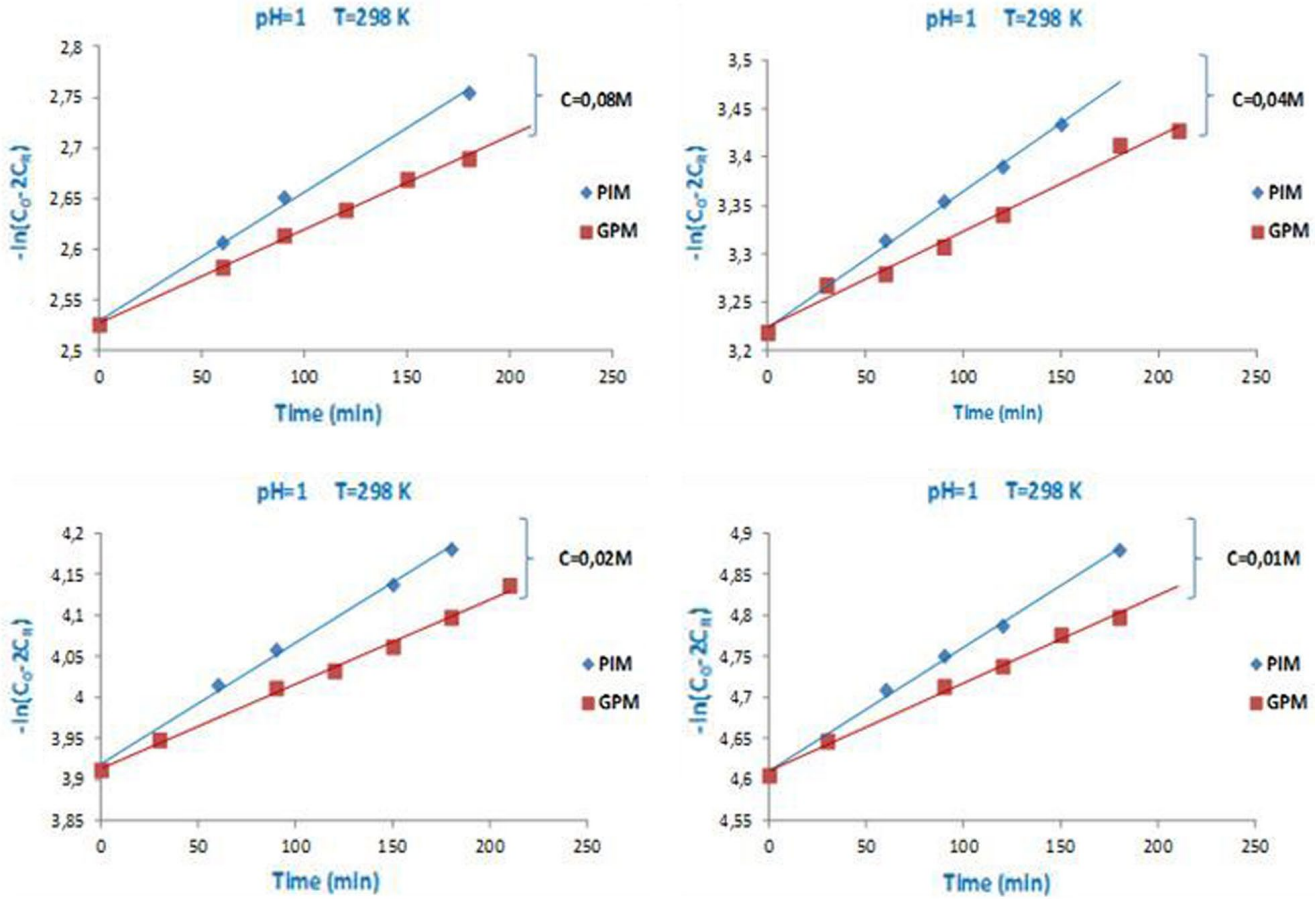
Evolution of the kinetic function − Ln (C_0_ − 2C_R_) = f (t) relative to the paracetamol extraction through the developed membranes at different concentrations C_0_, pH = 1 and T = 298 K

In this section, we have examined the effect of **C**_**0**_ on the evolution of macroscopic parameters ***P*** and ***J***_**0**_ relative to the facilitated extraction processes of paracetamol through all the developed membranes. Indeed, we have studied the processes at different **C**_**0**_: **0.08**, **0.04**, **0.02**, and **0.01** (mol L^−1^) at pH = 1 and T = 298 K. At all concentrations, the kinetic model has been verified, and the function − Ln (**C**_**0**_ − 2**C**_**R**_) = f (**t**) generated straight lines (Fig. 5). The values of ***P*** and ***J***_**0**_ were determined from the slopes of the straight lines (according to the expressions in Eqs. 3 and 4), presented in Table 1.

**Table 1:** Evolution of *P* and *J*_0_ parameters for extraction oriented processes of the paracetamol substrate at T = 298 K

Membrane type	PIM		GPM	
C_0_ initial concentration (mol L^−1^)	*P* * 10^7^ (cm^2^ s^−1^)	*J*_0_ * 10^5^ (mmol s^−1^ cm^−2^)	*P* * 10^7^ (cm^2^ s^−1^)	*J*_0_ * 10^5^ (mmol s^−1^ cm^−2^)
0.08	18.112	0.636	9.374	0.462
0.04	20.235	0.355	10.053	0.248
0.02	20.962	0.184	10.458	0.129
0.01	21.446	0.094	10.851	0.067

Analysis of the results grouped in Table 1 demonstrates that the used membranes are effective for paracetamol extraction. Based on the obtained values of macroscopic parameters (***P*** and ***J***_**0**_), the PIM membrane was more efficient than the GPM counterpart. However, it was noticed that the permeability ***P*** of the adopted membranes varies inversely with the initial paracetamol concentration in the feed phase C_0_, and an increase in the substrate concentration leads to a decrease in the parameter ***P***. However, the initial flux of paracetamol (***J***_**0**_) through each of the membranes increases with the substrate concentration C_0_. This reason can explain this is that during facilitated extraction of the substrate across the membrane, the association/dissociation mechanism of paracetamol with the extractive agent is faster when the initial substrate concentration is higher. This is due to the high difference in concentration between the feed and receiving phase (concentration gradient). Moreover, the results obtained for ***P*** indicate that this parameter is influenced by the competition of the substrate molecules to diffuse through the membrane phase. This evolution of the values of the parameters ***P*** and ***J***_***0***_ related to this oriented process has been observed and indicated by some previous works for similar processes related to the extraction of some organic compounds and metal ions [64–67].

### Acidity factor influence on the evolution of paracetamol extraction processes

**Fig. 6:**
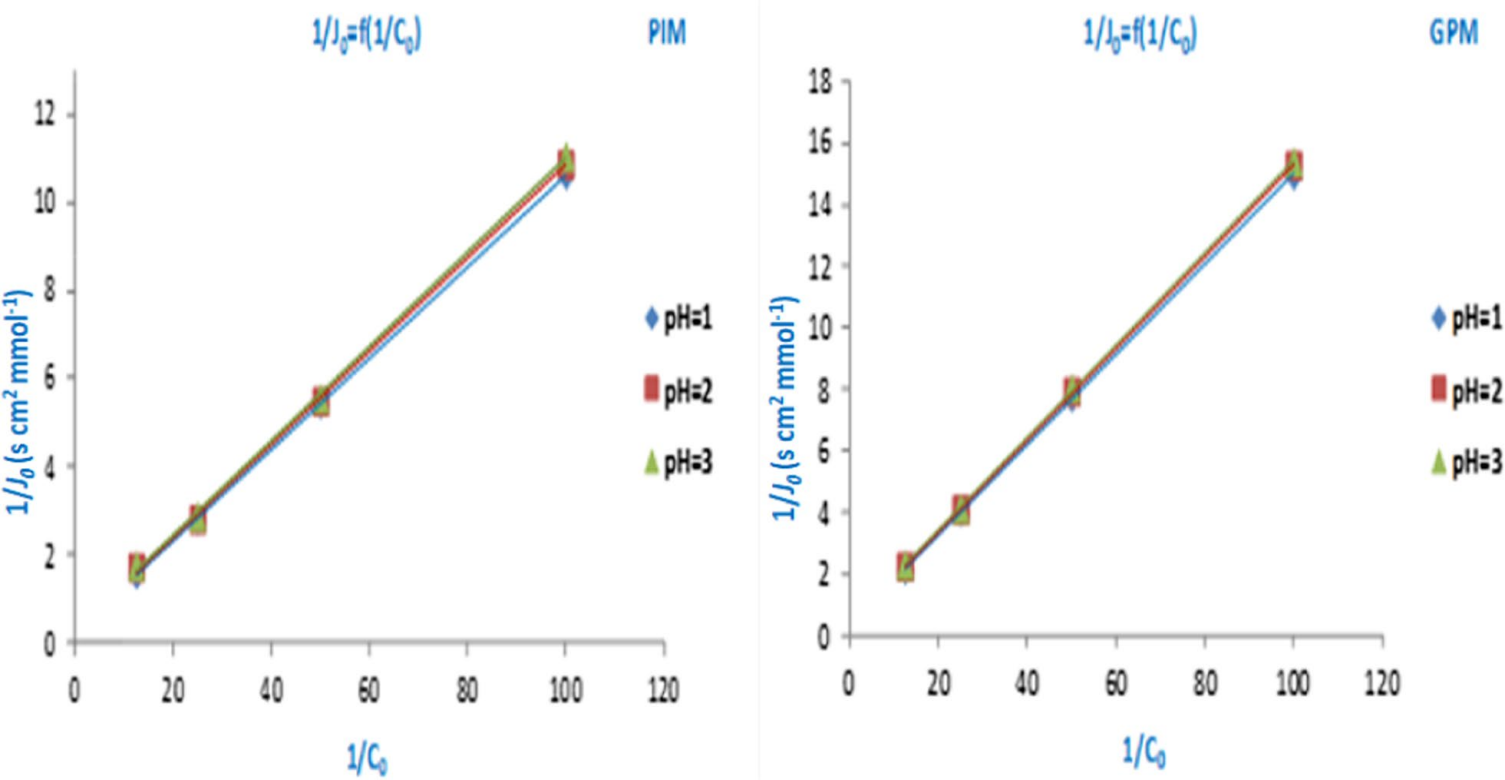
Lineweaver–Burk representations (1/*J*_0_ = f (1/C_0_)) for facilitated extraction processes across PIM and GPM membranes

**Fig. 7:**
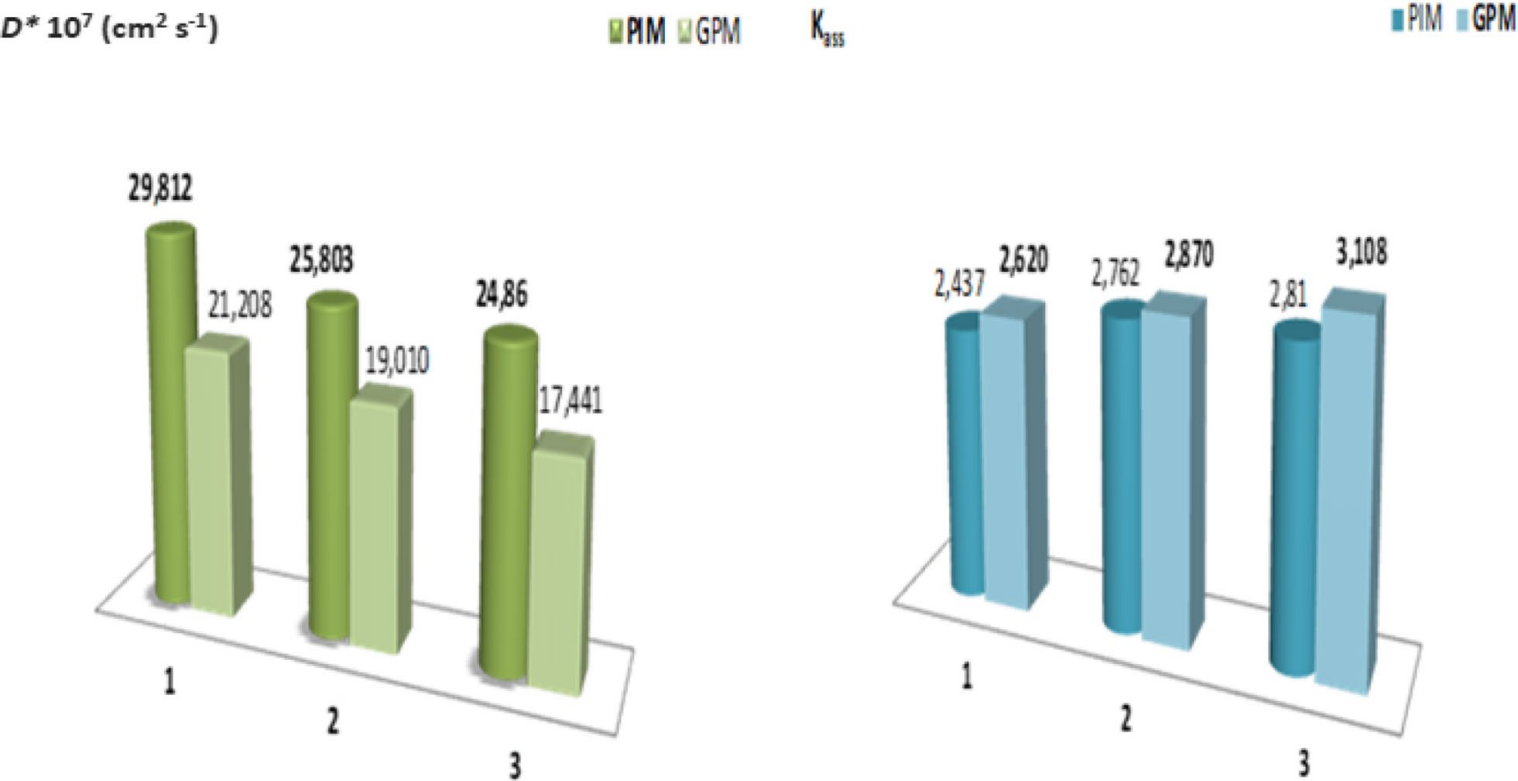
Influences acidity on the evolution of D* and *K*_*ass*_ parameters for the paracetamol extraction through the developed PIM and GPM membranes

To investigate the effect of acidity (feed and receiving aqueous solutions) on extraction efficiency through the adopted membranes, a series of experiments were performed at different pH (**1**, **2**, and **3**). Different substrate concentrations (0.01–0.08 mol L^−1^) were used for the experiments. The values of the macroscopic parameters ***P*** and ***J***_**0**_ were determined at each pH value (Table 2). The Lineweaver–Burk (L–B) representation 1/***J***_**0**_ = f (1/**C**_**0**_) was plotted using the values of initial fluxes. The slopes and intercepts of the straight lines are shown in Fig. 6. The D^*^ and ***K***_***ass***_ values (microscopic parameters) were estimated. The results are presented as histograms in Fig. 7.

**Table 2:** Evolution of *P* and *J*_0_ with pH during the extraction of paracetamol at T = 298 K

pH	C_0_ (mol L^−1^)	PIM	GPM		
		*P* * 10^7^ (cm^2^ s^−1^)	*J*_*0*_ *10^5^ (mmol s^−1^ cm^−2^)	*P* * 10^7^ (cm^2^ s^−1^)	*J*_*0*_ * 10^5^ (mmol s^−1^ cm^−2^)
1	0.08	18.112	0.635	9.373	0.462
	0.04	20.235	0.355	10.053	0.248
	0.02	20.962	0.184	10.458	0.129
	0.01	21.446	0.094	10.851	0.067
2	0.08	16.444	0.577	8.929	0.440
	0.04	20.178	0.354	9.866	0.243
	0.02	20.734	0.182	10.224	0.126
	0.01	20.962	0.092	10.625	0.065
3	0.08	16.159	0.567	8.713	0.429
	0.04	19.693	0.345	9.771	0.241
	0.02	20.491	0.180	10.080	0.124
	0.01	20.620	0.090	10.536	0.065

According to the results grouped in Table 2, it is clear that the pH of the aqueous solutions does not significantly influence the extraction oriented processes of paracetamol. On the other hand, it has been confirmed that the performance of the **PIM** membrane is better than that of the **GPM** counterpart at 3 acidic mediums.

As shown from Fig. 7, the apparent diffusion coefficient D* and association constant ***K***_***ass***_ vary inversely. The highest values of D* and the lowest ***K***_***ass***_ values are obtained for the most efficient membrane (**PIM**). These results explain the performances of the developed polymer membranes. The low values of ***K***_***ass***_ explain that the entity (Paracetamol-GA) in the membrane phase of PIM is less stable, which reflects by a higher diffusion in contrast to GPM. The high values of D* propose, firstly, that the diffusion of the substrate through the PIM was conditioned by successive interactions of substrate molecules with semi-mobile interaction sites of extractive agent in the membrane phase. Secondly, the passage of paracetamol through the GPM is a diffusion movement by successive jumps of substrate molecules from one site to another of fixed-sites of the extractive agent (Additional file 1: Fig. S3).

### Temperature influence on the evolution of oriented extraction processes of paracetamol

To confirm the previous results and determine the activation and thermodynamic parameters we examined the temperature factor influence on the evolution of the extraction process. The experiments were conducted at better acidity (**pH** = **1)**, **C**_**0**_ was varied in the range of 0.01 to 0.08 mol L^−1^ and at different temperatures (298, 303, and 308 K).

**Fig. 8:**
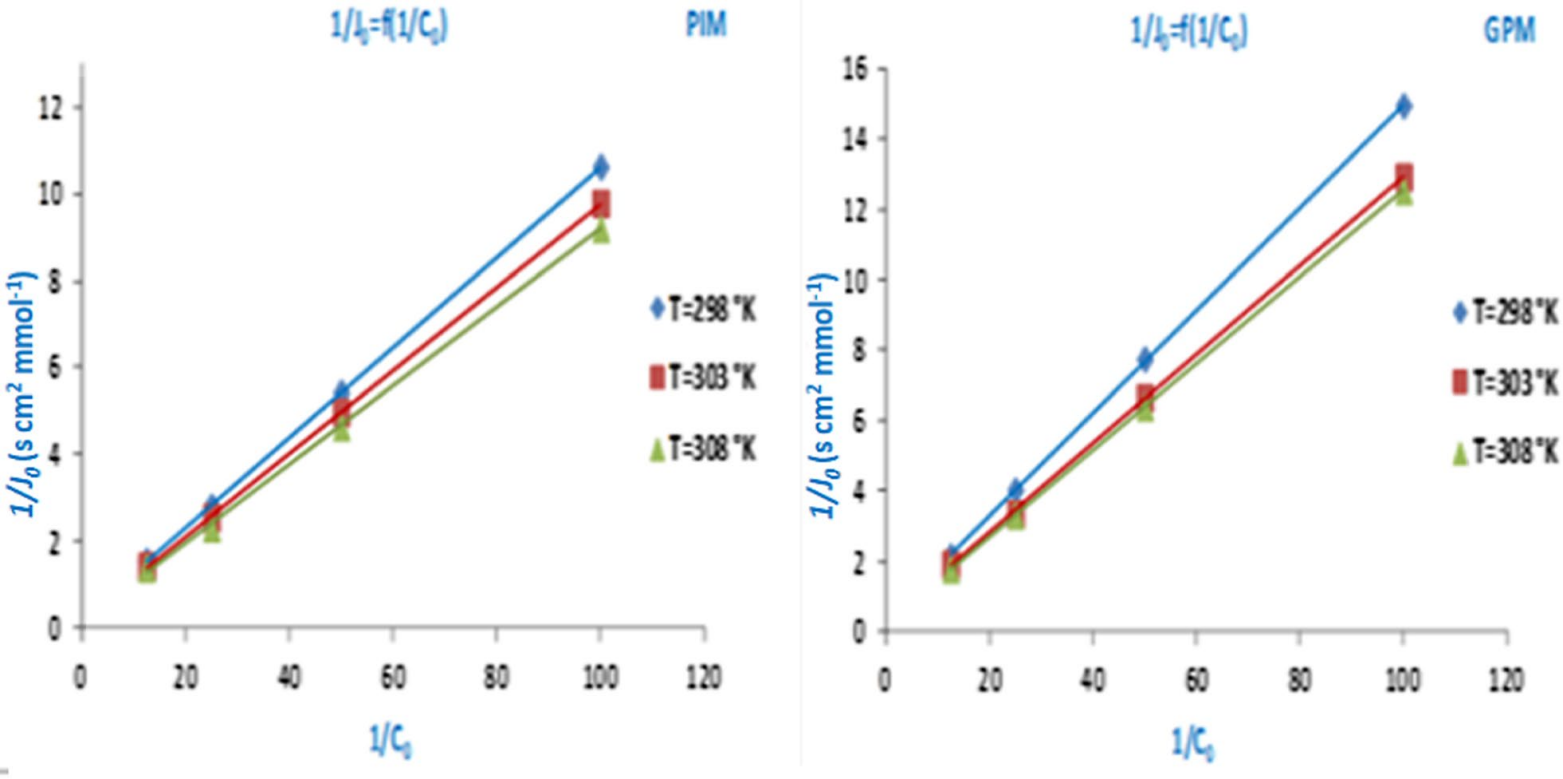
The L–B (1/*J*_0_ = f (1/C_0_)) for the oriented extraction processes of paracetamol through PIM and GPM membranes at different temperatures

The values of macroscopic parameters ***P*** and ***J***_**0**_ have been summarized in Table 3. The data reveals the impact of temperature on the facilitated extraction processes employed for paracetamol extraction. In addition, an increase in temperature leads to an increase in membrane performance. It was noted that the permeability and initial fluxes through the **PIM** membrane were higher than the permeability of the **GPM** membrane at all temperatures. To complete our study, we plotted the L–B curve (1*/****J***_**0**_ = f (1/**C**_**0**_)) (Fig. 8).

**Table 3:** Evolution of *P* and *J*_*0*_ parameters according to the medium temperature for extraction oriented processes of paracetamol

T (K)	C_0_ (mol L^−1^)	PIM		GPM	
		*P* * 10^7^ (cm^2^ s^−1^)	*J*_0_ * 10^5^ (mmol s^–1^ cm^−2^)	*P* * 10^7^ (cm^2^ s^−1^)	*J*_0_ * 10^5^ (mmol s^−1^ cm^−2^)
298	0.08	18.112	0.635	9.373	0.462
	0.04	20.235	0.355	10.053	0.248
	0.02	20.962	0.184	10.458	0.129
	0.01	21.446	0.094	10.851	0.067
303	0.08	19.751	0.693	10.465	0.516
	0.04	22.273	0.391	12.043	0.297
	0.02	22.886	0.201	12.189	0.150
	0.01	23.299	0.102	12.546	0.077
308	0.08	20.321	0.713	11.262	0.555
	0.04	24.410	0.428	12.200	0.301
	0.02	24.553	0.215	12.673	0.156
	0.01	24.738	0.108	12.934	0.080

**Fig. 9:**
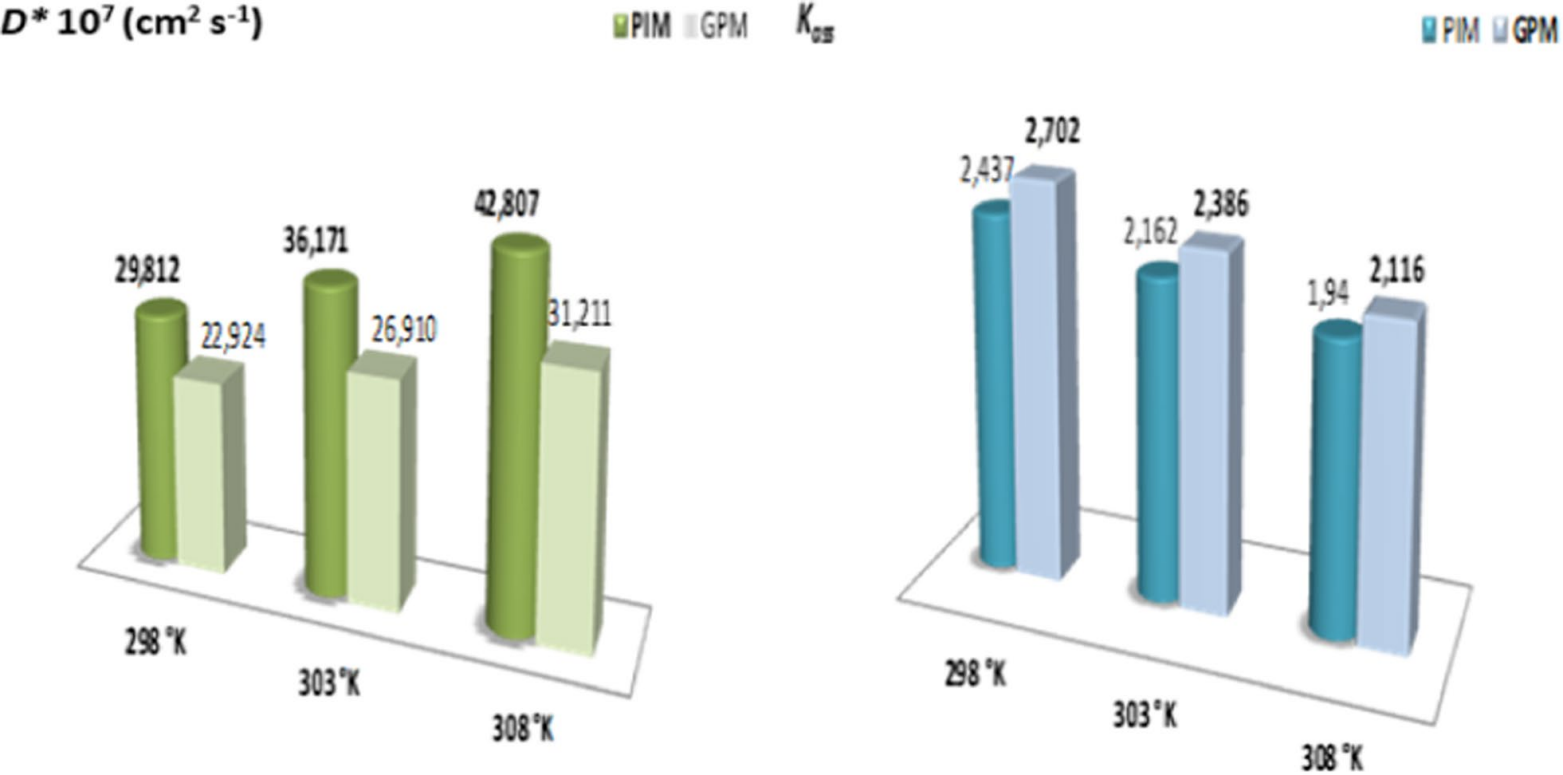
Evolution of specific D* and *K*_*ass*_ parameters according to the medium temperature for the facilitated extraction process of paracetamol through elaborated membranes

The linear evolution verified the adopted thermodynamic model and slopes and intercepts of the straight lines were used to determine the values of the apparent diffusion coefficient D* and the association constant ***K***_***ass***_. These two parameters are relative to the movement of the paracetamol molecules when they diffuse through each membrane. The values for these specific parameters and their evolution as a function of temperature are presented by the histograms in Fig. 9.

The results obtained for the microscopic parameters (Fig. 9) indicate the inverse evolution of ***K***_***ass***_ and D*. Therefore, confirming that an increase in the temperature leads to a decrease in stability of the ST entity formed in the membrane phase by interaction between substrate S and extractive agent T. Indeed, the low stability of the entity (**ST**) (translated by low values of ***K***_***ass***_) explain the faster substrate diffusion (**S**). The high D* and lower ***K***_***ass***_ values obtained at high temperatures can be potentially explained by improved membranes performance. The high values of apparent diffusion coefficient (D*), might indicate that the movement of paracetamol molecule across the organic phase of PIM and GPM membranes containing GA as an extractive agent is not pure diffusion.

In addition, according to the reviews and papers published by Hlaibi et al. [68, 69] related to the extraction of some organic compounds through SLM membranes types indicate identical evolutions for the specific parameters ***K***_***ass***_ and D* with a similar mechanism. Moreover, the values of ***K***_***ass***_ and D* parameters show that in the membrane phase, the interactions between molecules of organic compounds and extractive agent are low. In contrast, the values of apparent diffusion coefficient (D*) are high. At this step of the studies, we confirmed that the PIM membrane is more efficient than its counterpart GPM in terms of performance.

### Activation and thermodynamic parameters for the extraction studied processes

**Fig. 10:**
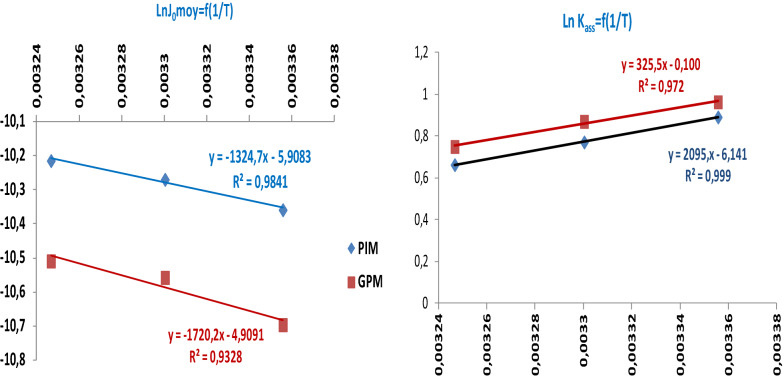
Evolution of Arrhenius and Van’t Hoff relationships for the paracetamol extraction processes through the PIM and GPM membranes

To elucidate the ***energetic*** or ***kinetic aspect*** that controls the mechanism of the studied processes, and to explain the performances of the prepared membranes, it is necessary to determine the values of the activation and thermodynamic parameters (***E***_***a***_, ***ΔH***^***≠***^_***ass***_, ***ΔS***^***≠***^, ***ΔH***^***≠***^_***diss***_, and ***ΔH***^***≠***^_***th***_) corresponding to the transition state of the substrate diffusion step across each organic membrane phase. For this, we have studied the evolution of ***J***_**0**_ and ***K***_***ass***_ values with temperature factor according to Arrhenius (Ln (***J***_**0*moy***_) = f (1/T)) and Van’t Hoff (Ln (***K***_***ass***_) = f (1/T) relationships (Eqs. 8 and 10) respectively (Fig. 10). The slopes and intercepts determined from the obtained straight line segments were used to determine the values of the activation and the thermodynamic parameters.

Table 4 presents the values of all the activation and thermodynamic parameters. Analysis of the activation parameters indicates that the transition state corresponding to the diffusion step requires little energy (***E***_***a***_ and ***ΔH***^***≠***^_***ass***_**)**. On the other hand, the negative activation entropy (***ΔS***^***≠***^) indicates that the transition state is perfectly ordered, depends on the substrate and extractive agent structures, and the orientation of their interaction sites. These results indicate that a favorable orientation of the interaction sites is required to achieve a good association between the paracetamol molecules and **GA** in the transition state with the bidentate sites (***ΔS***^***#***^ = − 300 J mol^−1^ K^−1^) (Additional file 1: Figs. S4 and S5). On the other hand, the low values of the important parameters (***ΔH***^***≠***^_***ass***_ and ***ΔH***^***≠***^_***diss***_) reveal the kinetic control aspect of the mechanisms of the oriented processes leading to good membrane performances even at low temperatures. This kinetic control aspect for the diffusion of paracetamol molecules through affinity polymer membranes can be described the structure of the molecules and the pharmacological and biological activities of these molecules that diffuse through cell membranes at a constant temperature.

**Table 4:** Activation and thermodynamic parameters corresponding to the transition state of the extraction process occurring through developed membranes

Activation parameters	*E*_*a*_ (kJ mol^−1^)	*ΔH*^*#*^_*ass*_ (kJ mol^−1^)	*ΔS*^*#*^ (J mol^−1^ K^−1^)	*ΔH*_*th*_ (kJ mol^−1^)	*ΔH*^*#*^_*diss*_ (kJ mol^−1^)
PIM	11.008	8.531	− 302.221	− 17.420	25.952
GPM	14.295	11.818	− 293.918	− 16.286	28.10

The very low values of (***Ea***, ***ΔH***^***≠***^_***ass***_, and ***ΔH***^***≠***^_***diss***_***)*** parameters relative to the facilitated extraction process across PIM–GA explain the good performance of this membrane type against to GPM–GA counterpart. Moreover, they confirm the influence of temperature factor and the inverse evolution of ***K***_***ass***_ and D* parameters. They also indicate that the substrate migration through the membrane phase was done by a mechanism of successive jumps of substrate molecules with semi-mobile interaction sites of the extractive agent in the PIM membrane phase. In contrast, the diffusion of paracetamol across the GPM is a movement of successive jumps from one site to another of the extractive agent fixed-sites. Indeed, several studies [28, 70, 71] confirmed these types of mechanisms in which the substrate moves while binding successively to several semi-mobile and fixed extractive agents (considered as a complexation site). Reversible association-dissociation reactions leading to the formation and decomposition of an unstable “host–guest” complex were carried out.

### Test for membrane stability

The stability test of elaborated PIM and GPM has been conducted under several conditions. The highest stability was observed at the tested pH and temperature during the extraction of paracetamol. The PVA and GPM membranes stability in an acidic medium was determined by repeating every 1–3 days at the end of the workday, an extraction of paracetamol was conducted in the same conditions. During every day, the membrane was also used for other experiments. The membrane was stable for about 6 months. This result is in good accordance with experiments described in the previous study [61, 70]. Moreover, after 6 months, the membranes were used for the same experiments without losing their effectiveness. They provided practically the same results as the obtained for the first experiment (A gap of 4.2% in the case of PIM and 3.8% for GPM). However, no degradation of membrane morphology occurred during the investigation. Therefore, it can be affirmed that the PIM and GPM membranes based on PVA and PSU with Gluconic acid as extractive agent manifested a stable characteristic with a good reproducibility during the proposed period. Additional file 1: Fig. S6 presented the evolution of the permeability relative to the facilitated extraction processes of paracetamol at C_0_ = 0.08 M, pH = 1 and T = 298 K, during a period of 6 months. Moreover, the membranes stability was also evaluated in terms of membrane mass change [72, 73]. Before and after the experiments, PIM and GPM membrane pieces were carefully weighed, and it was found a mass loss between 7 and 18% of the total weight for PIM and 5–11% for GPM. Hence, these results provide the use of these membranes since they preserve their performance features, such as low cost, and the possibility to prepare selective membranes, while providing the necessary stability to perform long-term experiments.

The membranes obtained after the extraction process were recovered and conditioned for SEM imaging. The observation from SEM shown in Additional file 1: Fig. S7 provides a qualitative view of the membrane morphology. The images obtained offer an idea of the stability of the PIM and GPM membranes after the extraction step. The almost similar morphology proves that the adopted membranes preserve the same characteristic before and after extraction experiments. Furthermore, concerning the PIM confirms that the relative swelling rate does not seem to influence its morphology.

## Conclusion

This work, conceptualized and quantified the performances and the mechanisms of oriented membrane processes for the facilitated extraction of paracetamol through PIM and GPM.

Two membranes PIM–GA and GPM–GA, were developed following the heat vulcanization and phase inversion methods and characterized by FT-IR and SEM techniques. The optimal identified operating conditions as substrate concentration, acidity, and temperature factors on the evolution of the different parameters were investigated, and the better paracetamol extraction was obtained for C_0_ = 0.01 mol L^−1^ at pH = 1 and 308 K, with D* (10^−7^ cm^2^ s^−1^) = 29.812 through PIM membrane.

Analysis of obtained results shows a good membrane performance observed even at low temperatures and indicates a kinetic aspect controlled the mechanism of extraction of this biologically active compound (paracetamol) through the two developed membranes. We can conclude that the paracetamol molecules can potentially diffuse through the cell walls having well-adapted structures at a constant temperature. Consequently, the kinetic control of the extraction processes of paracetamol is an original idea, and the studies produced logical results. It can be correlated to the molecular structures of paracetamol and the extractive agent. After these all studies, we can consider the adopted membranes would be very efficient for extracting and recovering paracetamol from industrial liquid discharges and providing a clean, sustainable, and environmentally friendly method for the extraction and recovery of the paracetamol molecule as a high-value substance.

## Supplementary Information

**Additional file 1: Figure S1.** Representation of the facilitated extraction cell. **Figure S2.** Swelling degree versus time of different membrane samples at pH = 1, 2 and 3. **Figure S3.** Mechanism of successive jumps on semi-mobile and fixed sites during the facilitated extraction process of paracetamol through the PIM–GA and GPM–GA membranes. **Figure S4.** Possible interaction sites between paracetamol and gluconic acid. **Figure S5.** Interaction sites between paracetamol and gluconic acid (Chemdrew). **Figure S6.** The permeability relative to the facilitated extraction processes of paracetamol at C_0_ = 0.08 M, pH = 1 and T = 298 K, during a period of six months. **Figure S7.** SEM micrographs after extraction process of (a, b) membrane cross-section (GPM–GA), (c d) membrane surface (PIM–GA).

## Data Availability

The datasets used and/or analyzed during the current study are available from the corresponding author on reasonable request.

## Abbreviations

[**T**]_**0**_: Concentration of carrier in the membrane phase (mol L^−^^1^)
C_0_: Initial concentration of paracetamol in the feed phase (mol L^−^^1^)
**C**_**R**_: Concentration of paracetamol in the receiving phase (mol L^−^^1^)
***P***: Permeability of the membrane (cm^2^ s^−^^1^)
***J***_**0**_: Initial flux (mmol s^−^^1^ cm^−^^2^)
***D****: Apparent diffusion coefficient (cm^2^ s^−^^1^)
***K***_***ass***_: Association constant
***l***: Membrane thickness (µm)
**t**: Time (s)
**V**: Volume of the receiving phase (cm^3^)
**S**: Active area of the membrane (cm^2^)
**T**: Temperature (K)
**R**: Ideal gas constant (J mol^−^^1^ K^−^^1^)
***E***_***a***_: Activation energy (KJ mol^−^^1^)
***ΔH***^***≠***^_***ass***_: Activation association enthalpy (KJ mol^−^^1^)
***ΔH***^***≠***^_***diss***_: Activation dissociation enthalpy (KJ mol^−^^1^)
***ΔS***^***≠***^: Activation entropy (KJ mol^−^^1^ K^−^^1^)
***ΔH***_***th***_: Thermodynamic enthalpy (KJ mol^−^^1^)
