# Supplementary material for: Kinetic control concept for the diffusion processes of paracetamol active molecules across affinity polymer membranes from acidic solutions
Source: BMC Chem. 2022 Jan 13;16(1):2. doi: 10.1186/s13065-021-00794-7 (PMC8759197; doi:10.1186/s13065-021-00794-7)
Supplement: Supplementary file 1 — Additional file 1: Figure S1. Representation of the facilitated extraction cell. Figure S2. Swelling degree versus time of different membrane samples at pH = 1, 2 and 3. Figure S3. Mechanism of successive jumps on semi-mobile and fixed sites during the facilitated extraction process of paracetamol through the PIM–GA and GPM–GA membranes. Figure S4. Possible interaction sites between paracetamol and gluconic acid. Figure S5. Interaction sites between paracetamol and gluconic acid (Chemdrew). Figure S6. The permeability relative to the facilitated extraction processes of paracetamol at C0 = 0.08 M, pH = 1 and T = 298 K, during a period of six months. Figure S7. SEM micrographs after extraction process of (a, b) membrane cross-section (GPM–GA), (c d) membrane surface (PIM–GA). [file 13065_2021_794_MOESM1_ESM.docx]

**Additional file**

**Kinetic control concept for the diffusion processes of Paracetamol active molecules across affinity polymer membranes from acidic solutions.**

**Sanae Tarhouchi^1*^, Rkia Louafy^1^, El Houssine. EL Atmani^1^, Miloudi Hlaïbi^1^**

*^1^Laboratoire Génie des Matériaux pour Environnement et Valorisation (GeMEV), Faculté des Sciences Ain Chock PB 5366, Maârif, Hasssan II University of Casablanca (UH2C), Maroc*.

**
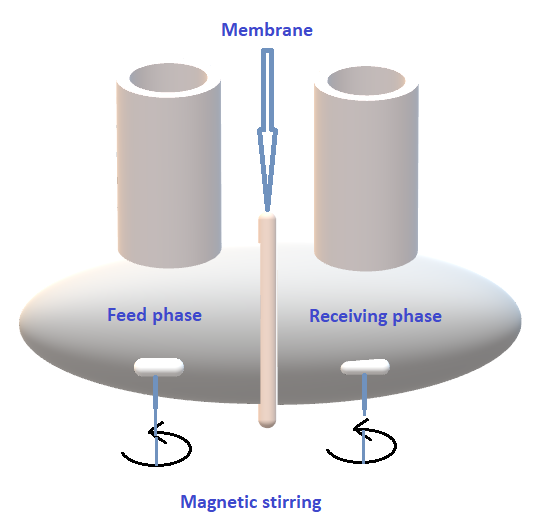
**

**Figure S1.** Representation of the facilitated extraction cell

**
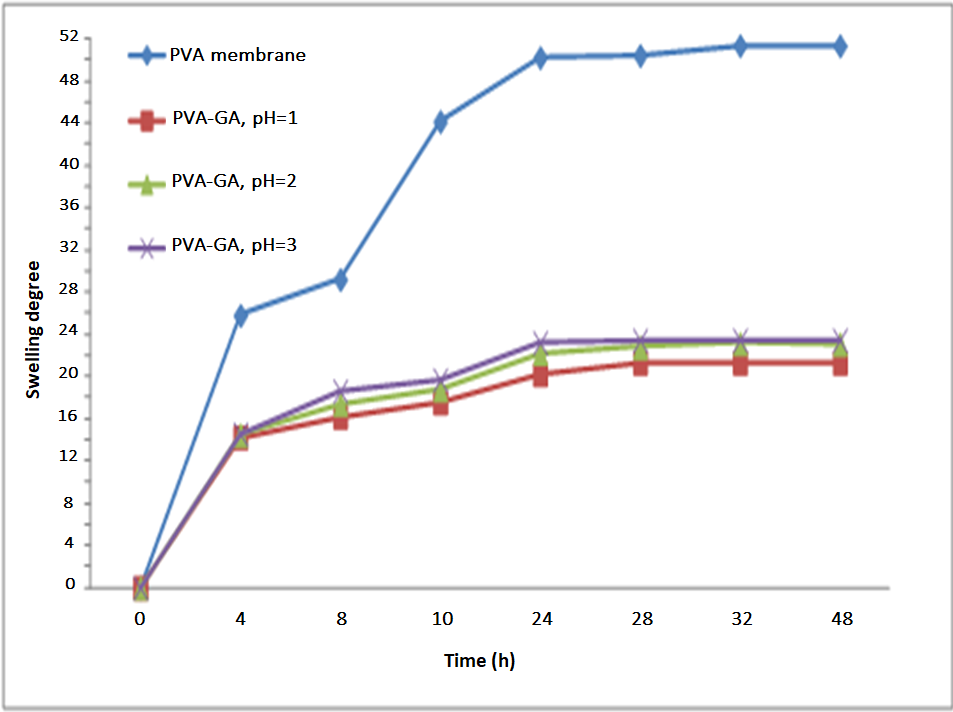
**

**Figure S2.** Swelling degree versus time of different membrane samples at pH=1, 2 and 3

**
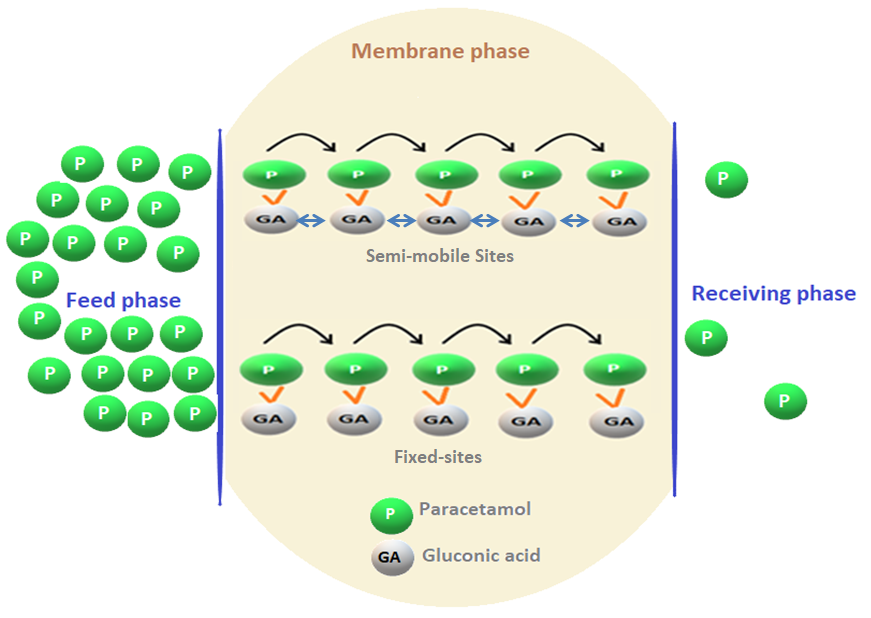
**

**Figure S3.** Mechanism of successive jumps on semi-mobile and fixed sites during the facilitated extraction process of paracetamol through the PIM-GA and GPM-GA membranes

**
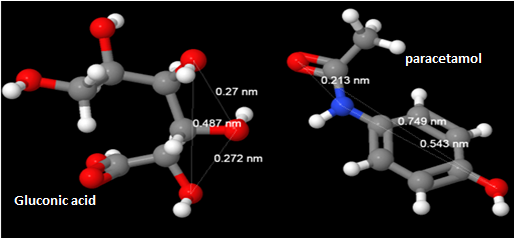
**

**Figure S4.** Possible interaction sites between paracetamol and gluconic acid

**
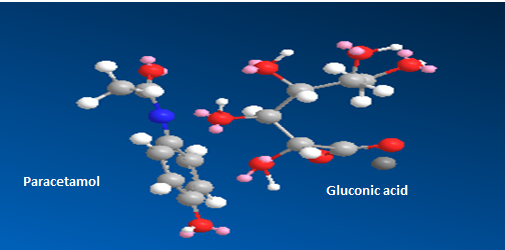
**

**Figure S5.** Interaction sites between paracetamol and gluconic acid (Chemdrew)

**Figure S6.** The permeability relative to the facilitated extraction processes of paracetamol at C_0_=0.08M, pH=1 and T=298 K, during a period of six months


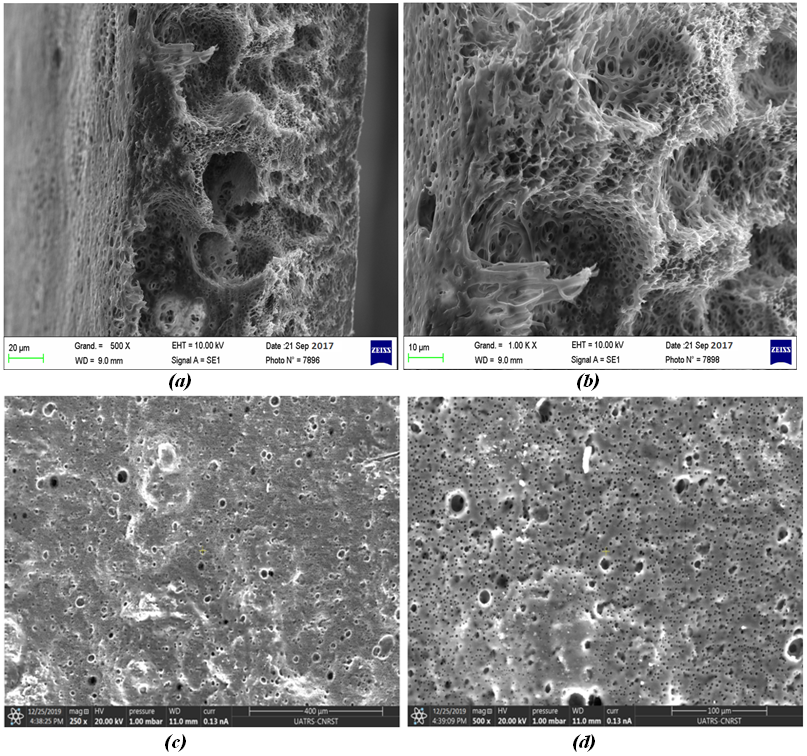


**Figure S7.** SEM micrographs after extraction process of (a,b) membrane cross-section (GPM-GA), (c,d) membrane surface (PIM-GA)
